# A 40-Marker Panel for High Dimensional Characterization of Cancer Immune Microenvironments by Imaging Mass Cytometry

**DOI:** 10.3389/fimmu.2019.02534

**Published:** 2019-10-29

**Authors:** Marieke E. Ijsselsteijn, Ruud van der Breggen, Arantza Farina Sarasqueta, Frits Koning, Noel F. C. C. de Miranda

**Affiliations:** ^1^Department of Pathology, Leiden University Medical Center, Leiden, Netherlands; ^2^Department of Immunohematology and Blood Transfusion, Leiden University Medical Center, Leiden, Netherlands

**Keywords:** imaging mass cytometry, cancer microenvironment, immunophenotyping, CyTOF, cancer immunity, immunotherapy

## Abstract

Multiplex immunophenotyping technologies are indispensable for a deeper understanding of biological systems. Until recently, high-dimensional cellular analyses implied the loss of tissue context as they were mostly performed in single-cell suspensions. The advent of imaging mass cytometry introduced the possibility to simultaneously detect a multitude of cellular markers in tissue sections. This technique can be applied to various tissue sources including snap-frozen and formalin-fixed, paraffin-embedded (FFPE) tissues. However, a number of methodological challenges must be overcome when developing large antibody panels in order to preserve signal intensity and specificity of antigen detection. We report the development of a 40-marker panel for imaging mass cytometry on FFPE tissues with a particular focus on the study of cancer immune microenvironments. It comprises a variety of immune cell markers including lineage and activation markers as well as surrogates of cancer cell states and tissue-specific markers (e.g., stroma, epithelium, vessels) for cellular contextualization within the tissue. Importantly, we developed an optimized workflow for maximum antibody performance by separating antibodies into two distinct incubation steps, at different temperatures and incubation times, shown to significantly improve immunodetection. Furthermore, we provide insight into the antibody validation process and discuss why some antibodies and/or cellular markers are not compatible with the technique. This work is aimed at supporting the implementation of imaging mass cytometry in other laboratories by describing methodological procedures in detail. Furthermore, the panel described here is an excellent immune monitoring tool that can be readily applied in the context of cancer research.

## Introduction

Technologies that support the high dimensional analysis of biological systems are essential in scientific research and have become increasingly relevant in clinical contexts. For instance, the advent of T cell checkpoint blockade therapies for cancer treatment has revitalized the field of cancer immunotherapy but also introduced an urgent need for the discovery of biomarkers that guide patient selection for therapies (1, 2). Furthermore, recent works making use of single-cell platforms based on RNA sequencing and mass cytometry have delivered a wealth of data revealing previously unappreciated cell subsets and novel functionalities (3–5). Nevertheless, most immunophenotyping techniques are held back by the lack of spatial resolution, limitations in the number of targets that can be visualized simultaneously, or cumbersome protocols. Methodologies such as flow cytometry can be employed to analyze multiple markers but are insufficient to chart the vast spectrum of immune cells in an unbiased manner (6). Single-cell mass cytometry overcomes this limitation by currently allowing the simultaneous analysis of ~40 cellular markers. However, it also lacks spatial information, failing to reveal tissue context and cellular interactions which are extremely relevant in physiological and disease states (7–9). Conversely, multispectral fluorescence imaging provides spatial context but is limited to few markers and is thus best suited to investigate specific research questions in large cohorts (10, 11). The recent introduction of imaging mass cytometry has considerably advanced the potential to simultaneously obtain information on phenotypes, their localization within a tissue, and to map cellular interactions.

Mass cytometry makes use of metal isotopes conjugated to antibodies of interest, in contrast to flow cytometry and immunofluorescence techniques that rely on fluorescent dyes. The metal isotopes are distinguished by mass in a time-of-flight mass spectrometer and, thus, the number of markers that can be detected simultaneously is not limited by spectral overlap. Since its discovery in 2009 (12), mass cytometry has been successfully applied for the immunophenotyping of cancer microenvironments. This has accelerated the discovery of new immune cell subsets, the assessment of potential biomarkers and correlation of immune-phenotypical changes to therapeutic outcomes (5, 13–15). Imaging mass cytometry makes use of a high resolution laser that is coupled to the mass cytometer (16). Successive ablations of small portions of tissue (~1 μm^2^) are analyzed by CyTOF (Cytometry Time-Of-Flight) thereby quantifying the presence of metal isotopes per area of tissue. This data is reconstructed into an artificial multilayer image resulting in a broad and comprehensive overview of protein expression *in situ*. Imaging mass cytometry can be employed for imaging up to 40 markers in different tissue sources (e.g., snap-frozen, FFPE), but the combination of a large number of antibodies in the same experiment raises methodological challenges: (1) The testing and validation of a large number of antibodies is an onerous and labor-intensive process. (2) The choice of tissue source must weigh the availability of antibodies directed against native or denatured antigen conformations. Furthermore, the use of FFPE requires that all antibodies function under the same antigen retrieval conditions. (3) The optimal immunodetection conditions are variable for different antibodies. By combining 40 antibodies into one experiment an optimized workflow must be designed in order to obtain best antibody performance.

We developed a 40 marker panel for the analysis of FFPE tissues by imaging mass cytometry. Next to a large amount of lineage and functional immune cell markers, the panel also contains surrogates of cancer cell states (e.g., proliferation, apoptosis) and structural markers (e.g., epithelium, stroma, vessels) for a comprehensive overview of cancer immune microenvironments but also to investigate cancer-immune cell interactions. Furthermore, we created an optimized immunodetection protocol in which antibodies are split into two incubation steps, thereby reducing the concentration of total antibody per working-solution and employing the optimal incubation time and temperature for each antibody. This work provides the scientific community with a ready-to-use imaging mass cytometry antibody panel and provides a blueprint for laboratories that wish to develop dedicated imaging mass cytometry panels.

## Methods

### Tissue Material

FFPE blocks were obtained from the department of Pathology of the Leiden University Medical Centre (Leiden, The Netherlands). Samples were anonymized and handled according to the medical ethical guidelines described in the Code of Conduct for Proper Secondary Use of Human Tissue of the Dutch Federation of Biomedical Scientific Societies. Both tonsil and colorectal cancer tissues were cut into 4 μm sections and placed on silane-coated glass slides (VWR, Radnor, PA, USA) for downstream analyses.

### Immunohistochemistry

Antibody specificity prior and post metal-conjugation as well as optimal antigen retrieval conditions were assessed by chromogenic immunohistochemistry (IHC). Tissue sections were deparaffinized and rehydrated with xylene and decreasing concentrations of ethanol, respectively, followed by endogenous peroxidase blockade using a 0.3% hydrogen peroxidase/methanol solution (Merck Millipore, Burlington, MA, USA). Sections were boiled in either Tris-EDTA (10 mM/1 mM, pH 9) or citrate (10 mM, pH 6) buffers for antigen retrieval and were allowed to cool down to room temperature. To decrease non-specific antibody binding, tissue sections were blocked with Superblock solution (Thermo Fisher Scientific, Waltham, MA, USA) and incubated overnight at 4°C with a primary antibody (Table 1). Following washes in PBS, the tissues were incubated with Poly-horseradish peroxidase solution (Immunologic, Duiven, The Netherlands) for 1 h at room temperature. Antibody binding was detected with DAB+ chromogen (DAKO, Agilent technologies, Santa Clara, Ca, USA) and the sections were counterstained with hematoxylin (Thermo Fisher Scientific).

**Table 1:** Forty marker FFPE panel for imaging mass cytometry.

			**Incubation**		
**Target**	**Clone**	**Metal**	**Time**	**Temperature**	**Dilution**
CD45	D9M8I	^89^Y	Overnight	4°C	50
CD39^**^	A1	^112^Cd/^114^Cd	Overnight	4°C	50
β-Catenin	D10A8	^115^In	Overnight	4°C	100
HLA-DR	TAL-1B5	^141^Pr	5 h	RT	100^*^
CD20	H1	^142^Nd	Overnight	4°C	100
CD68	D4B9C	^143^Nd	Overnight	4°C	100^*^
CD11b	D6X1N	^144^Nd	5 h	RT	100
CD4	EPR6855	^145^Nd	5 h	RT	50
CD8α	D8A8Y	^146^Nd	5 h	RT	50
CD31	89C2	^147^Sm	Overnight	4°C	100^*^
CD73	D7F9A	^148^Nd	5 h	RT	100
TGFβ	TB21	^149^Sm	5 h	RT	100
Granzyme B	496B	^150^Nd	Overnight	4°C	100^*^
CD57	HNK-1/Leu-7	^151^Eu	Overnight	4°C	100^*^
Ki-67	8D5	^152^Sm	Overnight	4°C	100^*^
CD3	D7A6E	^153^Eu	Overnight	4°C	50
TIM-3	D5D5R	^154^Sm	5 h	RT	100
LAG3	D2G4O	^155^Gd	5 h	RT	50
PD-L1	E1L3N	^156^Gd	Overnight	4°C	50
VISTA	D1L2G	^158^Gd	5 h	RT	100
FoxP3	D6O8R	^159^Tb	Overnight	4°C	50
PD-1	D4W2J	^160^Gd	5 h	RT	50
ICOS	D1K2T	^161^Dy	5 h	RT	50
IDO	D5J4E	^162^Dy	Overnight	4°C	100
CD14	D7A2T	^163^Dy	5 h	RT	100
CD204	J5HTR3	^164^Dy	5 h	RT	50
CD45RO	UCHL1	^165^Ho	Overnight	4°C	100^*^
D2-40	D2-40	^166^Er	Overnight	4°C	100^*^
CD56	EPR2566	^167^Er	Overnight	4°C	100
CD103	EPR4166(2)	^168^Er	5 h	RT	50
CD38	EPR4106	^169^Tm	Overnight	4°C	100^*^
T-bet	4B10	^170^Er	5 h	RT	50
CD15	BRA-4F1	^171^Yb	Overnight	4°C	100^*^
Cleaved Caspase-3	5A1E	^172^Yb	5 h	RT	100
CD163	EPR14643-36	^173^Yb	5 h	RT	50
CD7	EPR4242	^174^Yb	5 h	RT	100
P16 INK4A	D3W8G	^175^Yb	Overnight	4°C	100
CD11c	EP1347Y	^176^Yb	5 h	RT	100
Vimentin	D21H3	^194^Pt	Overnight	4°C	50
Pan-Keratin	AE1/AE3 and C11	^198^Pt	Overnight	4°C	50

* *These dilutions were applied in already diluted stock solutions as described in the methods section*.

** *CD39 is detected indirectly with a Qdot800 secondary antibody*.

### Antibodies and Metal Conjugation

Carrier-free IgG antibodies (concentrations between 0.5 and 1 mg/mL) were conjugated to purified lanthanide metals (Fluidigm, San Francisco, CA, USA) (Table 1) using the MaxPar antibody labeling kit and protocol (Fluidigm). After conjugation, all antibodies were eluted in 50 μl W-buffer (Fluidigm) and 50 μl antibody stabilizer (Candor Bioscience, Wangen im Allgäu, Germany) supplemented with 0.05% sodium azide. To conjugate anti-CD45 (clone D9M8I) to ^89^Y, Yttrium(III) chloride (Sigma-aldrich, Saint Louis, MO, USA) was dissolved in L-buffer (Fluidigm) to 1 M. Five microliters of a 50 mM working solution were used for conjugation as described in the MaxPar antibody labeling protocol. Conjugation of anti-Vimentin and anti-Keratin antibodies to ^194^Pt and ^198^Pt (Fluidigm), respectively, was performed as described previously by Mei et al. (17). In order to exclude that the labeling process substantially affected the performance of the antibodies, these were tested by IHC and immunodetection patterns were compared to their non-conjugated counterparts. Stock solutions of antibodies with a strong signal were further diluted in antibody stabilizer supplemented with 0.05% sodium azide. Antibodies were stored at 4°C and remained stable for at least 6 months.

### Imaging Mass Cytometry Acquisition

Prior to acquisition, the Hyperion mass cytometry system (Fluidigm) was autotuned using a 3-element tuning slide (Fluidigm) according to the tuning protocol provided by the Hyperion imaging system user guide (Fluidigm). As an extra threshold for successful tuning, a detection of at least 1,500 mean duals of ^175^Lu was used. Regions of interest were selected based on hematoxylin and eosin stains performed on consecutive tissue sections after which areas of 1,000 × 1,000 μm were ablated and acquired at 200 Hz. Ablation of one area took ~2 h. Data was exported as MCD files and visualized using the Fluidigm MCD^TM^ viewer. In order to better separate antibody signal and noise, each marker was visually inspected and a minimum signal threshold of 1 or 2 dual counts was set in the Fluidigm MCD^TM^ viewer.

## Stepwise Procedure for Immunodetection by Imaging Mass Cytometry

### Materials

– 4 μm tissue sections on silane-coated glass slides
– Xylene
– Ethanol (100, 70, 50%)
– 10x Antigen retrieval solution—low pH (pH 6, Thermo Fisher Scientific)
– Superblock solution
– PBS-TB (PBS supplemented with 0.05% Tween and 1% BSA)
– Metal-conjugated antibodies (Table 1)
– QDot800-labeled anti-mouse secondary antibody (Thermo Fisher Scientific)
– Intercalator-Ir (125 μM, Fluidigm)
– Demi-water
– 1.5 mL microtubes
– Pipettes (200, 10 μl)
– Pipette tips (200, 10 μl)
– Incubation chamber (humid, 4°C and room temperature)
– Microwave

### Day 1

1. Start with 4 μm FFPE sections on silane-coated glass slides
2. Deparaffinize tissue sections by incubating three times for 5 min in 100% xylene
3. Rinse tissue sections twice in 100% ethanol
4. Wash 5 min in ethanol (100%)
5. Rehydrate sections by rinsing in 70 and 50% ethanol
6. Dilute 10x antigen retrieval solution in demi-water
7. Preheat the 1x antigen retrieval solution for 10 min in a microwave
8. Rinse sections in unheated 1x antigen retrieval solution
9. Boil the sections in the preheated antigen retrieval solution for 10 min in the microwave
10. Remove excess buffer and allow the sections to cool down to room temperature for ~1 h
11. Rinse the sections with PBS-TB and incubate for 30 min with 200 μl Superblock solution
12. Prepare the anti-CD39 antibody (Mouse IgG1) by diluting it 1:50 in PBS-TB
13. Tap off excess Superblock solution and add 100 μl of the anti-CD39 antibody solution to each tissue section
14. Incubate the sections overnight at 4°C in a humid chamber

### Day 2

15. Wash the sections three times for 5 min with PBS-TB solution
16. Dilute the Qdot800-labeled, anti-mouse secondary antibody 1:50 in PBS-TB
17. Incubate the sections for 1 h at room temperature with 100 μl of Qdot800-labeled antibody solution
18. Wash the sections three times for 5 min with PBS-TB
19. Prepare the antibody mix for the 5 h room temperature incubation by diluting the antibodies in PBS-TB as described in Table 1
20. Add 100 μl of antibody mix to each section and incubate for 5 h at room temperature in a humid chamber
21. Wash the sections three times for 5 min with PBS-TB
22. Prepare the antibody mix for the overnight 4°C incubation by diluting the antibodies in PBS-TB as described in Table 1
23. Add 100 μl of antibody mix to each section and incubate overnight at 4°C in a humid chamber

### Day 3

24. Wash the sections three times for 5 min with PBS-TB
25. Dilute the Intercalator Ir 1:100 in PBS-TB
26. Incubate sections for 5 min at room temperature with 100 μl of diluted intercalator Ir
27. Wash the sections two times for 5 min with PBS-TB
28. Wash the sections 5 min with demi-water
29. Dry the slides under an air flow for 5 min and store at room temperature until ablation

### Timing

Day 1: Tissue preparation and incubation primary antibody (2 h)
  1 h hands on time
  Overnight incubation

Day 2: Incubation Qdot800-labeled secondary and two times incubation antibody mix (8.5 h)
  30 min hands on time
  2 h incubation
  30 min hands on time
  5 h incubation
  30 min hands on time
  Overnight incubation

Day 3: DNA stain and drying of tissue (30 min)
  30 min hands on time

### Notes

– All steps should be performed using plastic containers and without the use of glass to reduce metal binding to glass and metal contamination.
– Small differences exist in metal concentrations between batches. Thus, it should be taken into account that with a new metal-conjugation, optimal antibody dilutions can vary. Therefore, it is advised to validate the antibody performance by imaging mass cytometry after every conjugation.

## Results

To develop the 40 marker imaging mass cytometry antibody panel, antibody performance was assessed by IHC. Initially, 52 cellular targets, of interest for the field of cancer immunology, were selected for potential implementation in the panel. Antibody selection was based on in-house knowledge of antibody performance in IHC or, when unavailable, manufacturer's datasheets. All antibodies were tested with either low (pH 6) or high (pH 9) pH antigen retrieval buffers, with the former resulting in optimal antigen detection for the majority of antibodies. In total, 65 antibodies were tested for implementation into the panel of which 58 performed well in low pH antigen retrieval conditions (Supplementary Tables 1, 2).

**Figure 1:**
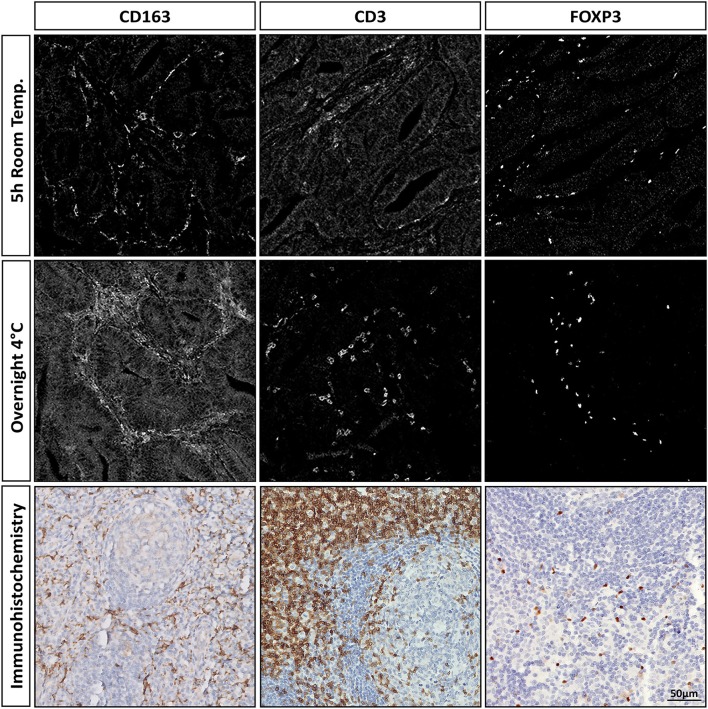
Comparison of antibody performance between two immunodetection incubation conditions for imaging mass cytometry and IHC. Incubation with all antibodies was either performed for 5 h at room temperature or overnight at 4°C for imaging mass cytometry. The markers CD163, CD3, and FOXP3 are representative for the variations observed by changing incubation time and temperature.

After antibody conjugation to their respective metals, imaging mass cytometry was performed on tissue sections that were incubated overnight at 4°C with an antibody mix containing all 40 antibodies (Table 1). It was observed that the immunodetection patterns were comparable between IHC and imaging mass cytometry confirming that IHC is a useful tool for the low cost validation of imaging mass cytometry antibodies. However, low signal intensity and/or high background were observed for a relevant proportion of the tested antibodies, when compared to IHC (e.g., anti-CD163, clone EPR14643-36, Figure 1). We reasoned that this was potentially due to the lack of a signal amplification step in the procedure or the excessive complexity of the antibody mix. Therefore, we tested different conditions for each antibody. For this, each antibody was incubated either overnight at 4°C or at room temperature for 5 h after which signal intensity and specificity were assessed by imaging mass cytometry and immunodetection patterns were compared to IHC (Figure 1, Supplementary Figure 1). For the majority of antibodies, striking differences in antibody performance (e.g., intensity, background) were observed between conditions. For instance, anti-CD163 (clone EPR14643-36) performed best after a 5 h incubation at room temperature, while anti-CD3 (clone D7A6E) performed optimally when incubated overnight at 4°C, as shown by its specific signal and low background when compared to a 5 h incubation at room temperature (Figure 1). In general, lowly abundant antigens were difficult to detect when their respective antibodies were incubated at 4°C and were best assessed by a room temperature incubation. In contrast, antibodies targeting abundant proteins lost specificity when incubated at room temperature which was resolved by their incubation at 4°C. However, there were exceptions to this pattern as demonstrated for the anti-CD163 antibody (clone EPR14643-36). After determining the optimal conditions for each antibody, they were assigned into one of two consecutive incubation steps as described by the stepwise protocol in the previous section. Furthermore, after determining the optimal dilution for each antibody, a number of antibody stock solutions (directly derived from the metal-labeling protocol) had to be diluted to allow their incorporation in the antibody mix volume. Specifically, anti-CD15, anti-CD31, anti-CD38, anti-CD45RO, and anti-Granzyme B were diluted 2 times, while anti-CD57, anti-D2-40, anti-HLA-DR, and anti-Ki-67 were diluted 3 times, and anti-CD68 was diluted 6 times.

A number of antibodies performed well in IHC and were, therefore, deemed good candidates for imaging mass cytometry but had to be excluded due to various reasons (Supplementary Table 2). As discussed, and in contrast to IHC, signal amplification is absent in imaging mass cytometry which limits the detection of low abundant markers. Thus, of the tested and conjugated antibodies, eight had to be excluded due to dim signal despite their good performance in IHC.

Conjugation of antibodies in house enabled the use of the ^89^Y, ^115^In, ^194^Pt, and ^198^Pt isotopes and allowed for easy implementation of new markers and clones. Of note, not all antibodies performed well after the conjugation protocol. This was observed for four antibodies that did not function in IHC after the conjugation procedure. Consequently, it was hypothesized that the binding domains of some antibodies or the antibody structure can be affected during the conjugation procedure which involves the partial reduction of antibodies. By making use of indirect antibody detection with a Qdot800-labeled secondary antibody containing ^112^Cd/^114^Cd isotopes, an additional marker could be included in the panel, resulting in a total of 40 markers.

**Figure 2:**
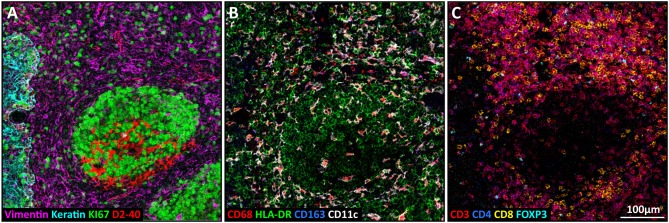
Detection of structural, myeloid and lymphoid markers in a single region by imaging mass cytometry on tonsil tissue. **(A)** Structural markers: Vimentin (purple), Ki-67 (green), D2-40 (red), and Keratin (cyan). **(B)** Myeloid markers: CD68 (red), CD163 (blue), HLA-DR (green), and CD11c (white). **(C)** Lymphoid markers: CD3 (red), CD8 (yellow), CD4 (blue), and FOXP3 (cyan).

**Figure 3:**
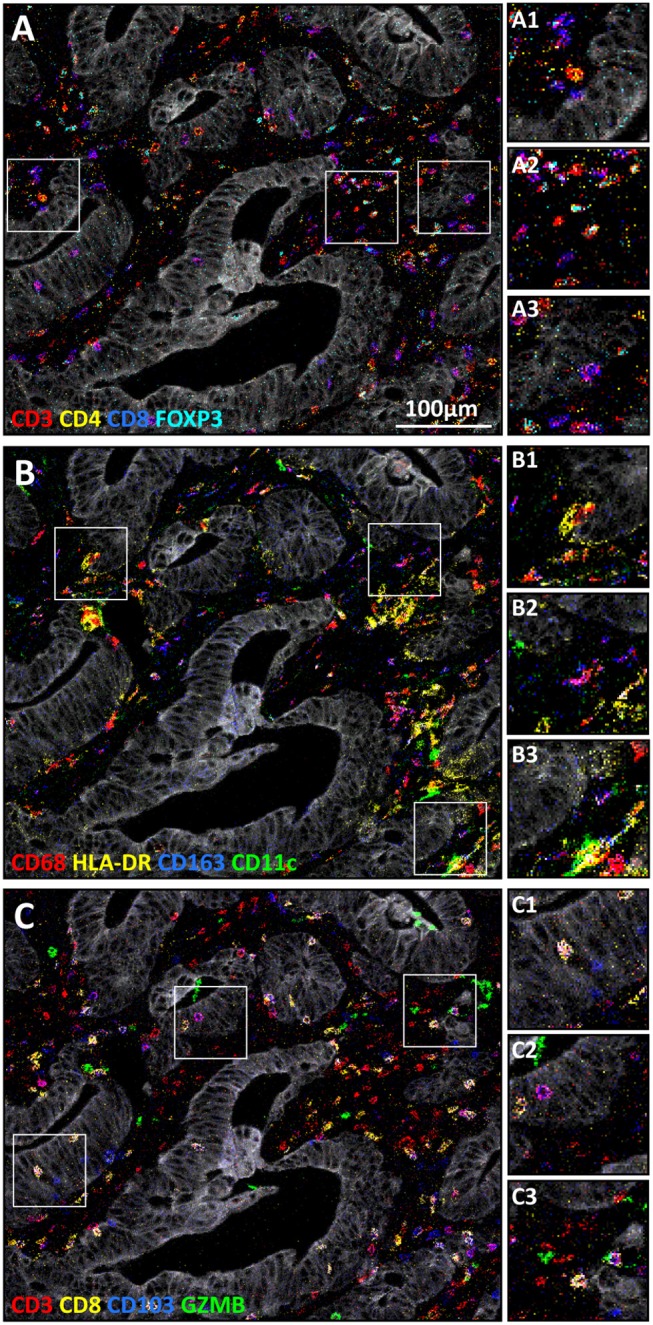
Visualization of immune cell types in a single region by imaging mass cytometry in colorectal cancer tissues. The tumor is marked by keratin (white) in **(A–C)**. The following cell types, amongst others, could be visualized: **(A)** T-helper cells (A1, CD3^+^CD4^+^), regulatory T cells (A2, CD3^+^FOXP3^+^), and cytotoxic T cells (A3, CD3^+^CD8^+^), **(B)** HLA-DR^+^ macrophages (B1, CD68^+^HLA-DR^+^), CD163^+^ macrophages (B2, CD68^+^CD163^+^), and CD11c^+^ macrophages (B3, CD68^+^CD11c^+^), **(C)** tissue resident cytotoxic T cells (C1, CD3^+^ CD8^+^CD103^+^), tissue resident T helper cells (C2, CD3^+^CD8^−^CD103^+^), and activated T cells (C3, CD3^+^GZMB^+^).

The here described 40 marker panel for imaging mass cytometry comprised tissue structural markers, myeloid and lymphoid lineage markers (Figure 2), but also proteins involved in immune activation, immune checkpoints, and surrogates of cellular states (Supplementary Figure 1). To demonstrate the applicability of the panel for extensive immunophenotyping of tissue, imaging mass cytometry was performed on colorectal cancer tissues and evaluated for a plethora of immune cells. In a single region of interest, among other cell types, tumor cells (Keratin^+^), T-helper cells (CD3^+^CD4^+^), cytotoxic T cells (CD3^+^CD8^+^), regulatory T cells (CD3^+^FOXP3^+^, Figure 3A), HLA-DR^+^ macrophages (CD68^+^HLA-DR^+^), CD163^+^ macrophages (CD68^+^CD163^+^), and CD11c^+^ macrophages (CD68^+^CD11c^+^, Figure 3B), were identified. Moreover, the panel allows for the visualization of additional features which are essential for the study of cancer immunology such as tissue residency-like (e.g., CD103^+^ T cells) and activation phenotypes (granzyme B^+^ T cells, Figure 3C).

## Discussion

We report a 40-antibody panel for imaging mass cytometry of FFPE tissues with focus on cancer immunology. Furthermore, we optimized a protocol for optimal performance of antibodies by making use of consecutive incubation steps at different duration and temperature.

Imaging mass cytometry is a low throughput technology that generates high dimensional data, applicable on both FFPE and snap-frozen tissue. FFPE tissue is readily available at the departments of Pathology as it constitutes the standard method for archiving tissues in most medical centers. Furthermore, FFPE tissue allows for the use of tissue micro arrays (TMA) which, as compared to whole slides, increases the throughput and reduces costs associated with imaging mass cytometry.

For single-cell mass cytometry users, expertise in flow cytometry is extremely useful for supporting the development of antibody panels. However, imaging mass cytometry on tissue has a closer resemblance to IHC or immunofluorescence detection methods as shown by the high comparability in antibody performance between these techniques. It is important to note that the lack of a signal amplification step in imaging mass cytometry procedures, as compared to IHC, can hamper the visualization of low abundant proteins, as it is the case for some immune checkpoint molecules. Therefore, it should be taken into account that cell populations with a dim expression of those markers are potentially not observed by imaging mass cytometry. A major prerequisite for analysis of FFPE tissues is the need of antigen retrieval, where the crosslinks created by formalin fixation are broken to make the epitopes accessible for antibody binding. Antigen retrieval is commonly done by boiling tissues in a buffer with a pH ranging from 3 to 10, which can greatly influence antibody performance (18). In an imaging mass cytometry panel all antibodies should perform with the same antigen retrieval protocol. In the design of the current panel it was observed that the majority of antibodies performed best when employing citrate (pH 6) as antigen retrieval buffer, in line with the manufacturers' recommendations for the majority of antibodies. Thus, for implementation in an IMC panel, it is recommended to select antibodies that are known to perform in IHC on FFPE tissue and to consider the recommended antigen retrieval conditions.

To allow the detection of 40 markers and ensure optimal performance of all antibodies, tissue incubation with the former was separated into three steps. First, tissues were incubated overnight at 4°C with a primary anti-CD39 antibody that was detected by a Qdot800-labeled, secondary antibody. Quantumdots contain the cadmium isotopes ^112^Cd and ^114^Cd, which can be detected by CyTOF (14, 19). Importantly, this indirect detection step can be employed for primary antibodies that are sensitive to labeling procedures. Subsequently, tissues were incubated with approximately half of the antibodies for 5 h at room temperature, followed by an overnight incubation with the remainder of the antibodies at 4°C. Because optimal antibody performance can vary greatly between conditions we propose that antibody performance is tested in both settings upon design of an imaging mass cytometry panel.

In the development of the described panel all antibodies were conjugated in house. With the limited number of commercially available FFPE validated conjugated antibodies, in house conjugation allows for easy adaptations to the panel. Furthermore, it enabled the use of the ^89^Y, ^115^In, ^194^Pt, and ^198^Pt isotopes and together with the use of Qdot800 (^112^Cd and ^114^Cd), 40 markers can be detected simultaneously.

Sixty-five antibodies/clones were initially tested to be employed in the current imaging approach. Four of those did not perform with low pH antigen retrieval buffer, four were destroyed upon metal conjugation and eight antibodies failed to detect low abundant markers. Two antibodies, anti-Histone H3 (clone D1H2) and anti-pSMAD2 (clone 138D4), were conjugated and detectable by imaging mass cytometry and can potentially be implemented in future panels.

Immunological approaches to cancer therapy have been on the rise and with this, multiplex immunophenotyping techniques have become essential in contexts of research and immunomonitoring. Previous techniques were limited in either the number of markers that can be analyzed simultaneously or by the lack of spatial information. This was overcome with the development of technologies like imaging mass cytometry. The panel and methodology described here can support the extensive immunophenotyping of cancer FFPE tissues. It was designed to provide a comprehensive characterization of the major immune cell subsets present in tissues, in relation to cancer cells. The in-depth study of cellular interactions and investigation of multicellular contexts of antitumor immune responses is supported by the inclusion of tissue structural markers, immune lineage markers but also markers that inform about the functional state of the different cell types. To our knowledge, few labs are currently operating with a 40 marker panel in imaging mass cytometry and, therefore, this work supports the maximization of the potential of imaging mass cytometry across research groups.

## Data Availability Statement

The datasets generated for this study are available on request to the corresponding author.

## Author Contributions

MI performed experiments and wrote the manuscript. RB performed antibody conjugation procedures. AF evaluated the immunohistochemistry results. FK supervised the study and revised the manuscript. NM initiated, supervised the study, and wrote the manuscript.

### Conflict of Interest

The authors declare that the research was conducted in the absence of any commercial or financial relationships that could be construed as a potential conflict of interest.

## References

[1] Havel JJ Chowell D Chan TA. The evolving landscape of biomarkers for checkpoint inhibitor immunotherapy. Nat Rev Cancer. (2019) 19:133–50. 10.1038/s41568-019-0116-x 30755690 PMC6705396

[2] de Miranda N Trajanoski Z. Advancing cancer immunotherapy: a vision for the field. Genome Med. (2019) 11:51. 10.1186/s13073-019-0662-6 31358048 PMC6661948

[3] Savas P Virassamy B Ye C Salim A Mintoff CP Caramia F Single-cell profiling of breast cancer T cells reveals a tissue-resident memory subset associated with improved prognosis. Nat Med. (2018) 24:986–93. 10.1038/s41591-018-0078-7 29942092

[4] Simoni Y Becht E Fehlings M Loh CY Koo SL Teng KWW. Bystander CD8(+) T cells are abundant and phenotypically distinct in human tumour infiltrates. Nature. (2018) 557:575–9. 10.1038/s41586-018-0130-2 29769722

[5] de Vries NL van Unen V Ijsselsteijn ME Abdelaal T van der Breggen R Farina Sarasqueta A. High-dimensional cytometric analysis of colorectal cancer reveals novel mediators of antitumour immunity. Gut. (2019). 10.1136/gutjnl-2019-318672. [Epub ahead of print]. 31270164 PMC7063399

[6] Newell EW Cheng Y. Mass cytometry: blessed with the curse of dimensionality. Nat Immunol. (2016) 17:890–5. 10.1038/ni.3485 27434000

[7] Galon J Costes A Sanchez-Cabo F Kirilovsky A Mlecnik B Lagorce-Pages C. Type, density, and location of immune cells within human colorectal tumors predict clinical outcome. Science. (2006) 313:1960–4. 10.1126/science.1129139 17008531

[8] Mahmoud SM Paish EC Powe DG Macmillan RD Grainge MJ Lee AH. Tumor-infiltrating CD8+ lymphocytes predict clinical outcome in breast cancer. J Clin Oncol. (2011) 29:1949–55. 10.1200/JCO.2010.30.5037 21483002

[9] Halama N Michel S Kloor M Zoernig I Benner A Spille A. Localization and density of immune cells in the invasive margin of human colorectal cancer liver metastases are prognostic for response to chemotherapy. Cancer Res. (2011) 71:5670–7. 10.1158/0008-5472.CAN-11-0268 21846824

[10] Parra ER Uraoka N Jiang M Cook P Gibbons D Forget MA. Validation of multiplex immunofluorescence panels using multispectral microscopy for immune-profiling of formalin-fixed and paraffin-embedded human tumor tissues. Sci Rep. (2017) 7:13380. 10.1038/s41598-017-13942-8 29042640 PMC5645415

[11] Ijsselsteijn ME Brouwer TP Abdulrahman Z Reidy E Ramalheiro A Heeren AM. Cancer immunophenotyping by seven-colour multispectral imaging without tyramide signal amplification. J Pathol Clin Res. (2019) 5:3–11. 10.1002/cjp2.113 30191683 PMC6317065

[12] Bandura DR Baranov VI Ornatsky OI Antonov A Kinach R Lou X. Mass cytometry: technique for real time single cell multitarget immunoassay based on inductively coupled plasma time-of-flight mass spectrometry. Anal Chem. (2009) 81:6813–22. 10.1021/ac901049w 19601617

[13] van Unen V Li N Molendijk I Temurhan M Hollt T van der Meulen-de Jong AE. Mass cytometry of the human mucosal immune system identifies tissue- and disease-associated immune subsets. Immunity. (2016) 44:1227–39. 10.1016/j.immuni.2016.04.014 27178470

[14] Bendall SC Simonds EF Qiu P Amir el AD Krutzik PO Finck R. Single-cell mass cytometry of differential immune and drug responses across a human hematopoietic continuum. Science. (2011) 332:687–96. 10.1126/science.1198704 21551058 PMC3273988

[15] Levine JH Simonds EF Bendall SC Davis KL Amir el AD Tadmor MD. Data-driven phenotypic dissection of AML reveals progenitor-like cells that correlate with prognosis. Cell. (2015) 162:184–97. 10.1016/j.cell.2015.05.047 26095251 PMC4508757

[16] Giesen C Wang HA Schapiro D Zivanovic N Jacobs A Hattendorf B. Highly multiplexed imaging of tumor tissues with subcellular resolution by mass cytometry. Nat Methods. (2014) 11:417–22. 10.1038/nmeth.2869 24584193

[17] Mei HE Leipold MD Maecker HT. Platinum-conjugated antibodies for application in mass cytometry. Cytometry A. (2016) 89:292–300. 10.1002/cyto.a.22778 26355391

[18] Shi SR Shi Y Taylor CR. Antigen retrieval immunohistochemistry: review and future prospects in research and diagnosis over two decades. J Histochem Cytochem. (2011) 59:13–32. 10.1369/jhc.2010.957191 21339172 PMC3201121

[19] Bjornson ZB Nolan GP Fantl WJ. Single-cell mass cytometry for analysis of immune system functional states. Curr Opin Immunol. (2013) 25:484–94. 10.1016/j.coi.2013.07.004 23999316 PMC3835664

