# Supplementary material for: A 40-Marker Panel for High Dimensional Characterization of Cancer Immune Microenvironments by Imaging Mass Cytometry
Source: Front Immunol. 2019 Oct 29;10:2534. doi: 10.3389/fimmu.2019.02534 (PMC6830340; doi:10.3389/fimmu.2019.02534)
Supplement: Supplementary file 1 [file Data_Sheet_1.docx]

***Supplementary figures***


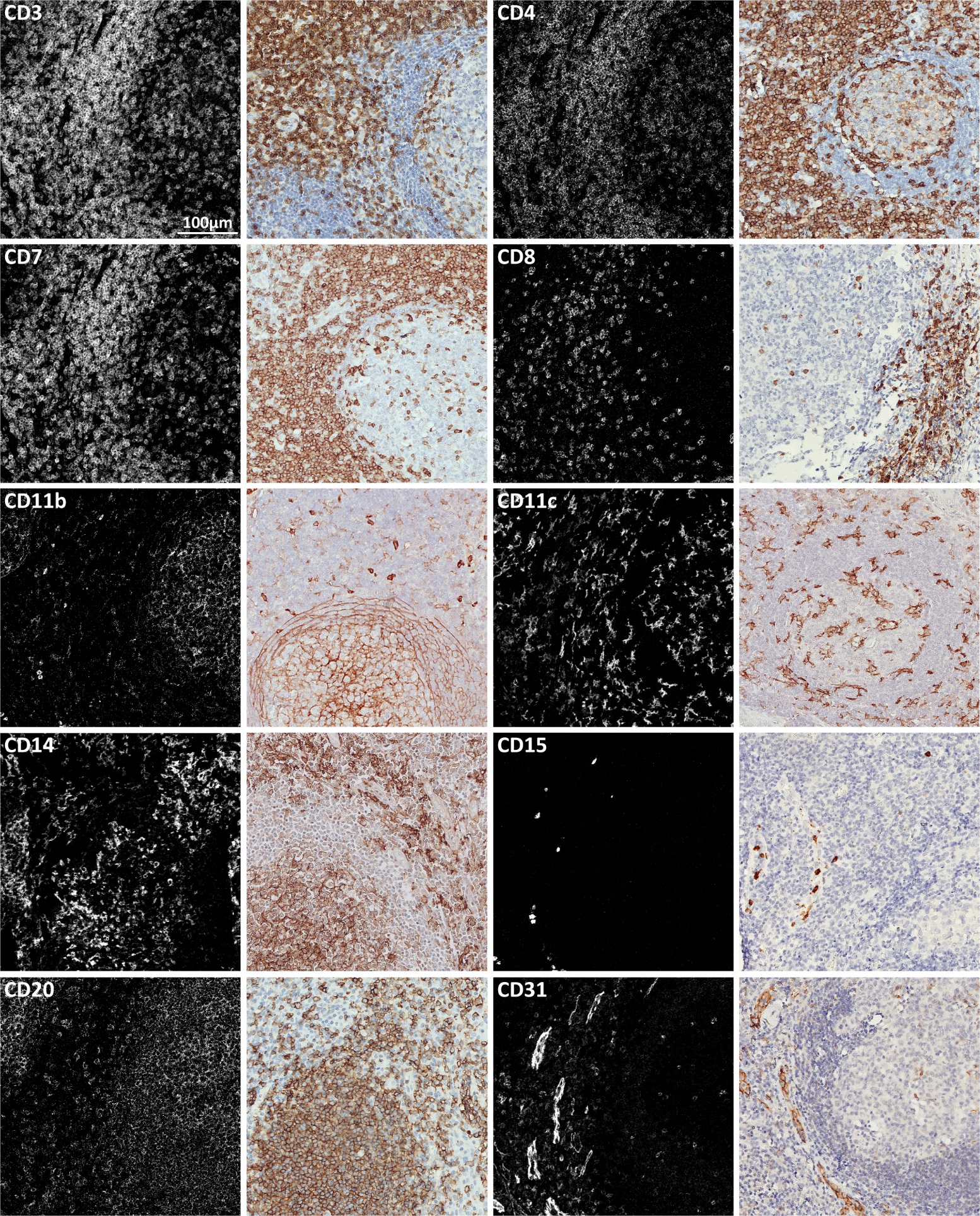


**Supplementary figure 1a.** Comparison of immunodetection patterns between imaging mass cytometry (left panels) and IHC (adjacent right panels) of the 40 markers included in the described imaging mass cytometry panel. Incubation of each antibody for imaging mass cytometry was performed at the determined optimal conditions. Both IHC and imaging mass cytometry were performed on tonsil tissue.


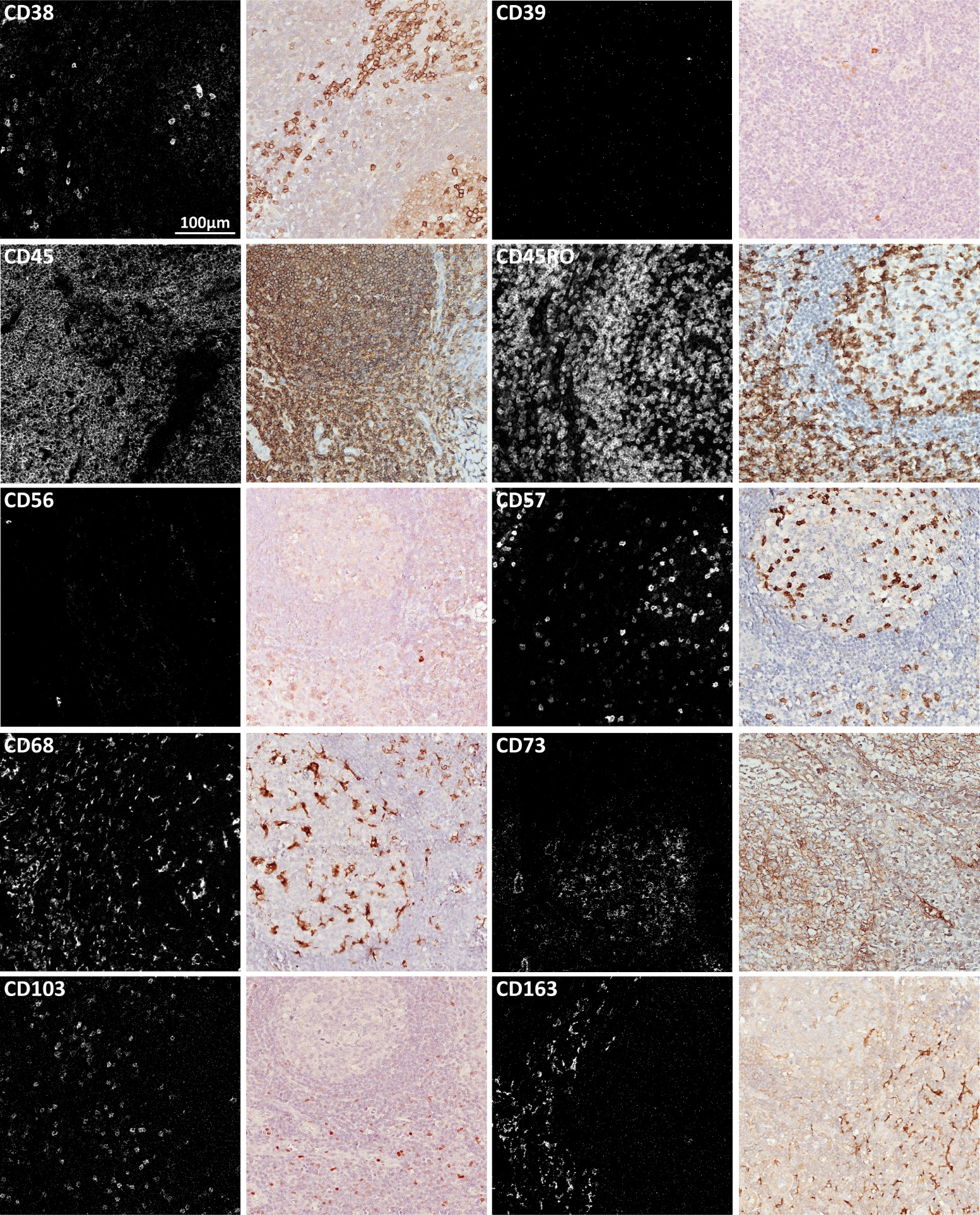


**Supplementary figure 1b.** Comparison of immunodetection patterns between imaging mass cytometry (left panels) and IHC (adjacent right panels) of the 40 markers included in the described imaging mass cytometry panel. Incubation of each antibody for imaging mass cytometry was performed at the determined optimal conditions. Both IHC and imaging mass cytometry were performed on tonsil tissue.

**
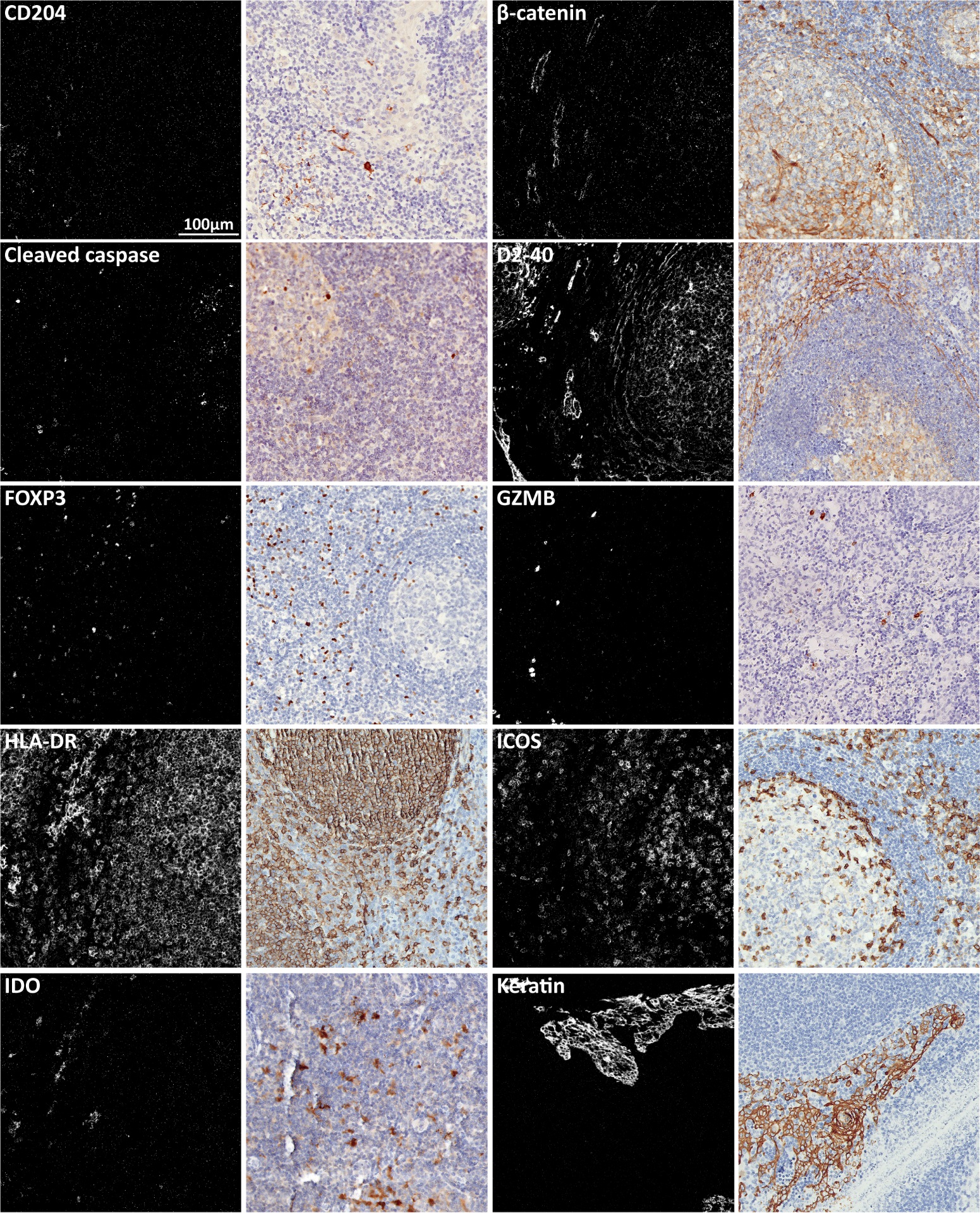
**

**Supplementary figure 1c.** Comparison of immunodetection patterns between imaging mass cytometry (left panels) and IHC (adjacent right panels) of the 40 markers included in the described imaging mass cytometry panel. Incubation of each antibody for imaging mass cytometry was performed at the determined optimal conditions. Both IHC and imaging mass cytometry were performed on tonsil tissue.

**
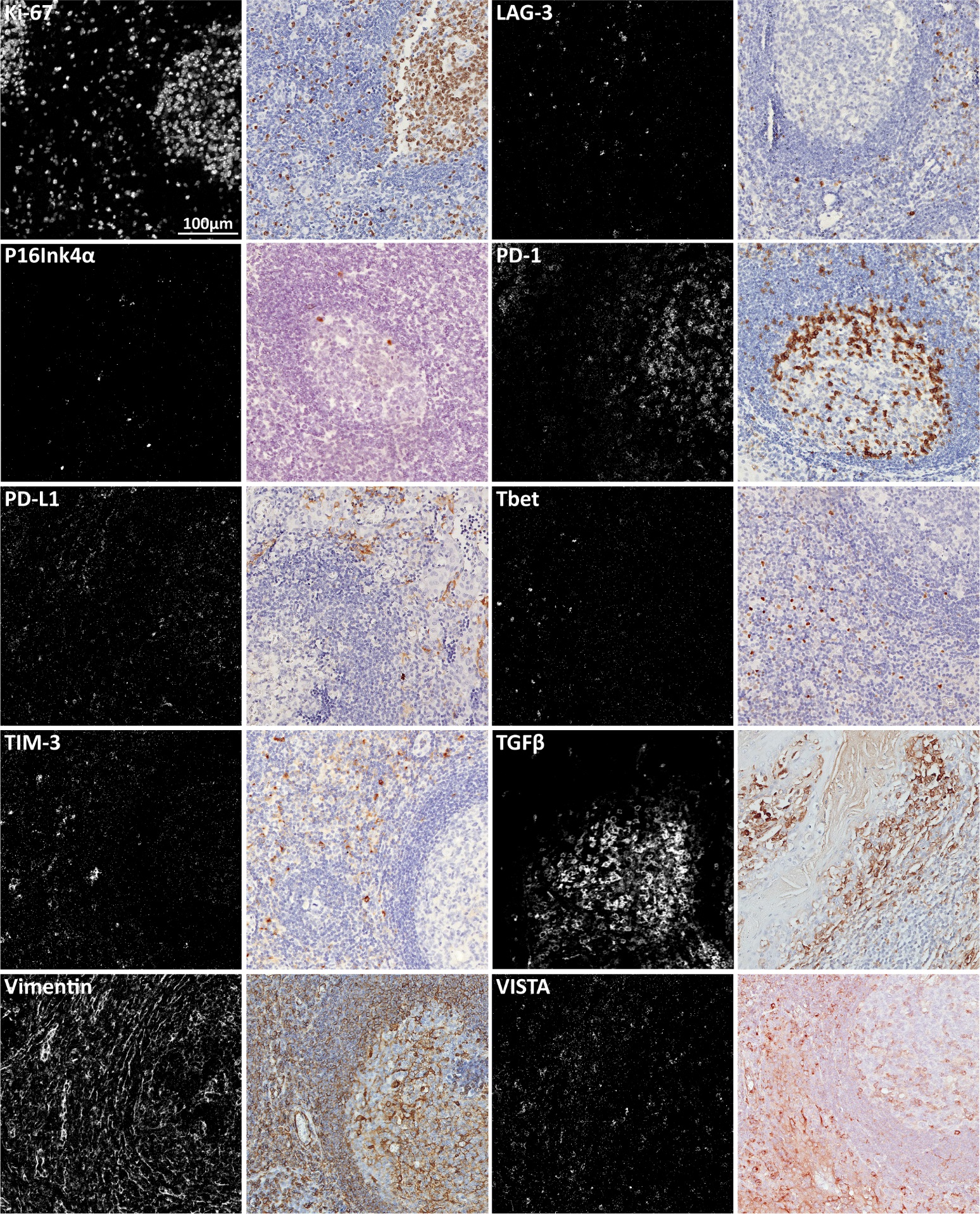
**

**Supplementary figure 1d.** Comparison of immunodetection patterns between imaging mass cytometry (left panels) and IHC (adjacent right panels) of the 40 markers included in the described imaging mass cytometry panel. Incubation of each antibody for imaging mass cytometry was performed at the determined optimal conditions. Both IHC and imaging mass cytometry were performed on tonsil tissue.

**Supplementary table 1.** Supplier and antigen retrieval (AR) information of 40-antibody imaging mass cytometry panel.

**Supplementary table 2.** Antibodies unsuitable for inclusion in the 40-antibody imaging mass cytometry panel and reasons for exclusion.
